# Maternal age differences in cognitive regulation: examination of associations and interactions between RSA and EEG frontoparietal alpha power coherence

**DOI:** 10.3389/fnhum.2023.1188820

**Published:** 2023-08-24

**Authors:** Jennifer D. Christensen, Martha Ann Bell, Kirby D. Deater-Deckard

**Affiliations:** ^1^Department of Psychological and Brain Sciences, University of Massachusetts Amherst, Amherst, MA, United States; ^2^Virginia Tech, Department of Psychology, Blacksburg, VA, United States

**Keywords:** age difference, development, vagal control, respiratory sinus arrhythmia, frontoparietal alpha power coherence, cognitive regulation, executive function

## Abstract

Strong cognitive regulation is advantageous for flexible, responsive parenting. Optimal cognitive regulation is reliant on associations between physiological mechanisms of central and peripheral nervous system functioning. Across middle adulthood there may be shifts in how cognitive regulation functions, reflecting changes in the associations and interactions between these physiological mechanisms. Two physiological indicators of cognitive regulation are autonomic regulation of the heart (e.g., respiratory sinus arrhythmia, RSA) and activity of the brain’s frontoparietal network (e.g., frontoparietal EEG alpha power coherence, FPc). In the current study we examined maternal age differences (*N* = 90, age *M* = 32.35 years, *SD* = 5.86 years) in correlations and interactions between RSA and FPc in the statistical prediction of cognitive regulation [i.e., executive function (EF), effortful control (EC), cognitive reappraisal (CR)]. Age-related patterns involving interaction between RSA and FPc were found, pointing to a potential shift from optimization to compensation for changes with aging or alternately, the effects of age-based decrements in functioning. Findings are discussed in the context of adult developmental changes in maternal caregiving.

## 1. Introduction

Sensitive and responsive parenting requires efficient and flexible cognitive regulation, which is a foundational feature of complex mental processing. Cognitive regulation allows for attending to salient information (and disregarding irrelevant information) and is reflected in optimized corresponding activity among various psychophysiological mechanisms in the central and peripheral nervous systems (Moilanen and Manuel, 2017; Yu et al., 2020; Han et al., 2021; McCurdy et al., 2022; Tellegen et al., 2022). Poorer cognitive regulation is associated with negative emotional states and reactive as well as dysregulated behavior and thoughts in parents themselves and their children (Bridgett et al., 2015; Giuliano et al., 2015; Evans et al., 2020; Lisitsa et al., 2021). Thus, it is important to understand the whole system of parental cognitive regulation involving the nervous system, cognitions, emotions and behaviors.

Parents’ cognitive regulation varies between individuals from day to day, but overall, these individual differences are quite stable across time and situations (Deater-Deckard, 2004; Bridgett et al., 2015). What is not yet known is whether these individual differences in neurobiological and behavioral components of cognitive regulation work together differently through development and aging across mid-life when parents are doing the bulk of their “heavy lifting” of childrearing. If found, such shifts may reflect aging-based shifts from optimization of neurocognitive resources to compensatory processes (derived from Selection, Optimization, Compensation Theory or SOC; Baltes, 1997; see also Amodio, 2010; Reuter-Lorenz and Park, 2010; Cabeza et al., 2018). Given this major gap in knowledge regarding adult development, we conducted a novel exploration of potential maternal age differences between two physiological indicators and cognitive regulation in mothers aged 21–49 years.

Cognitive regulation is a key aspect of behavioral and emotion regulation (Turner and Husman, 2008; Saarikallio, 2011; Zimmermann and Iwanski, 2014; Kakhki et al., 2022), and is defined broadly as attending to and utilizing salient and non-salient information which can then lead to optimal use of cognitive and physiological resources. In the current study, we operationalize cognitive regulation using three of the most widely used constructs that also have been shown to be pertinent to maternal and paternal caregiving behavior: executive function, effortful control, and cognitive reappraisal (Crandall et al., 2015; Zeytinoglu et al., 2017; Lin et al., 2022). Executive function encompasses at least three related classes of processes involving working memory, inhibitory control, and attention/set shifting that together result in controlled responses to the environment (Marcovitch et al., 2010; Miyake and Friedman, 2012). Effortful control is a similar construct that refers to the shifting focus and inhibiting impulsive responses as needed (Evans and Rothbart, 2007). Cognitive reappraisal involves reframing salient negative events and internal sensations in order to reduce their aversiveness (Gross, 1998). Together, these variables capture related, distinct aspects of cognitive regulation that aid in responsive and supportive (vs. reactive and punitive) parenting behaviors in the many situations that require flexibility in maternal attention and cognition, impulse control, and active updating of appraisals (Evans et al., 2020; Tellegen et al., 2022). It is well—vs. poorly—regulated caregiving behaviors that is of concern given their well-established effects on children’s and adolescent’s wellbeing and psychological health (Yu et al., 2020; Lisitsa et al., 2021).

Regarding developmental changes, the cognitive components of executive function and effortful control (i.e., inhibitory control, attentional control, working memory) show rapid growth across early childhood with continued, very gradual improvement across adolescence into young adulthood, followed by a gradual decrease across adulthood and accelerated decreases in old age (Rhodes, 2004; Rodriguez-Aranda and Martinussen, 2006; Rosselli and Torres, 2019; LaPlume et al., 2022). The developmental literature regarding cognitive reappraisal shows evidence of improvements with age across childhood and adolescence into early adulthood, but the findings regarding age-based changes across adulthood and into old age are mixed as to whether gradual decreases occur or not (McRae et al., 2012; Opitz et al., 2012; Hamilton and Allard, 2021). And like all cognitively demanding tasks, executive function, effortful control, and cognitive reappraisal show very substantial individual differences at all ages, even among the very young and very old.

Cognitive regulation is supported by cerebral and cardiovascular mechanisms including respiratory sinus arrhythmia (RSA) measured using electrocardiography (ECG) (Wang et al., 2013; Capuana et al., 2014; Balzarotti et al., 2017; Borges et al., 2020) as well as frontal and parietal brain region activity measured via alpha frequency power using encephalography (EEG) (Sauseng et al., 2005; van der Helden et al., 2010; Başar and Güntekin, 2012; Sadaghiani et al., 2012). RSA is a key indicator of autonomic regulation of the heart via the vagus nerve (Yasuma and Hayano, 2004; Porges, 2007). Higher RSA is indicative of better autonomic regulation. Developmentally, it increases in early childhood, plateaus across childhood through young adulthood, then gradually starts to decrease across adulthood as part of normative aging (Hrushesky et al., 1984; Giardino et al., 2003; Patriquin et al., 2015). There is robust support of the association between RSA and many aspects of cognitive regulation. For example, research indicates a positive correlation between higher RSA and better cognitive regulation (Geisler et al., 2013; Capuana et al., 2014). Higher RSA indicates more adaptability and efficient cardio physiological responsiveness to cognitive demands, in addition to more efficient and effective regulation of emotional states (Balzarotti et al., 2017).

Turning to the brain, EEG alpha power is one key indicator of brain activity patterns associated with cognitive regulation because it provides information about the degree of active effortful processing (vs. disengagement and passivity) in wakeful cognitive states. We utilized correlated activation and deactivation measurements in frontal and parietal regions—operationalized using EEG frontoparietal alpha-power coherence, which is the squared correlation of alpha-power values between frontal and parietal sites (Başar and Güntekin, 2012; Babaeeghazvini et al., 2021; Heugel et al., 2022; Warbrick, 2022). Covarying individual differences in power values representing activation and deactivation is thought to indicate the functional co-activation and co-deactivation of those brain regions; for the purposes of the current study, frontoparietal alpha-power coherence is thought to represent activity of the frontoparietal (also known as the central executive) network (Laufs et al., 2003; Heugel et al., 2022).

Encephalography frontoparietal alpha-power coherence is evident in early infancy but begins to decrease in childhood (Bell and Wolfe, 2007). The association between cognitive regulation and frontoparietal alpha-power coherence in early childhood indicates that there are cognitive function benefits to frontoparietal regions being synchronized (Bell, 2012). Beyond the few studies of infancy and childhood, little is known about age-related developmental changes in frontoparietal alpha-power coherence in adolescence and adulthood. By comparison, there is more research in those age periods that has examined fMRI coherence. Given that fMRI coherence and alpha-power coherence are inversely correlated in adolescence and adulthood (Goldman et al., 2002; Laufs et al., 2003), the fMRI coherence literature can provide some insights. fMRI frontoparietal coherence gradually increases across adolescence and early adulthood (Campbell et al., 2012; Cuevas et al., 2012; Thorpe et al., 2016; Solis et al., 2021). Its average levels across adulthood are not established, but some research indicates that fMRI coherence decreases in old age (Campbell et al., 2012). Thus, it stands to reason that EEG frontoparietal alpha-power coherence may decrease across adolescence and through early adulthood and increase among aging adults. These developmental age-related changes are thought to reflect maturation and learning, whereby cognitively demanding tasks become more automated as the frontoparietal network becomes more efficient at processing, but then become more correlated again to reflect increasing effortful processing to compensate for aging-related degradation in the system. Regarding individual differences, at any given age point, higher EEG frontoparietal alpha-power coherence likely reflects greater effort, and lower frontoparietal alpha-power coherence reflects recruitment of more physiological resources (Bell, 2001; Bell and Wolfe, 2007).

Of particular importance for the current study, major portions of the frontoparietal network play a key role in the neocortical regulation of the vagus nerve and cardiac activity that together comprehensively represent the central and peripheral nervous systems’ roles in cognitive regulation (Balzarotti et al., 2017; Holzman and Bridgett, 2017). The prefrontal cortex regions encompass—but are not limited to—portions of the insular cortex, the anterior cingulate, and the amygdala (Thayer and Lane, 2000; Thayer and Ruiz-Padial, 2002; Smith et al., 2017). The parietal regions we assessed in the current study have been identified as important regions involved in cognitive processing of the environment (Han et al., 2004). These regions are integral to self-regulation (Thayer and Lane, 2000; Thayer et al., 2009).

In the current study, we focused specifically on the most relevant neurophysiological, behavioral, and self-reported indicators of the cognitive regulation system for mothers caring for young children. Women’s transitions into parenthood and the hour by hour, day after day caregiving role place chronic and acute demands on their cognitive and physiological regulation (along with many other systems) for decades, typically spanning the 20 s through the 50 s (Deater-Deckard and Panneton, 2018; Mehta et al., 2020). This occurs within the context of normative developmental changes across adulthood in key aspects of cognitive regulation and physiology described above.

It is worth noting that in the current analysis, we do not include measures of parenting behavior. Some members of the current authorship team have previously published findings from the current study dataset, showing the patterns of covariation between individual differences in maternal harsh parenting behaviors (self-reported and observed), executive function, heart rate and EEG alpha-power reactivity values (Deater-Deckard and Bell, 2017). Specifically, modest to moderate increases in heart rate along with larger decreases in alpha-power (when going from a resting state to an active cognitive task state) yielded the strongest association between the highest executive function levels and lowest harsh parenting levels. However, that prior research did not consider maternal age differences, nor did it consider the roles of frontoparietal alpha-power coherence and RSA in accounting for the key indicators of self-regulation that are fundamental to non-reactive, regulated parenting behavior (e.g., executive function, effortful control, and cognitive reappraisal).

As all women with children develop and age across adulthood, they are experiencing developmental changes amidst parenting challenges to many systems of the body—including those supporting cognitive regulation. According to Selection, Optimization and Compensation (SOC) theory (Baltes, 1997; Baltes et al., 1999), these changes lead to a gradual shift from *optimization* (i.e., maximal efficacy and efficiency) of available capacities and resources in young adulthood, to *compensation* (i.e., adapting utilization to sustain efficacy in response to decreasing capacity). This theorized shift is not related to changes in the role demands on women, but rather reflects an adaptive response to developmental changes in regulatory capacities and resources that optimize cognitive regulation in young adult parents (i.e., in their 20 and 30 s) then shifts gradually toward compensation for sustaining cognitive regulation among older parents (i.e., in their 40 and 50 s). Reuter-Lorenz and Lustig (2005) suggested three potential mechanisms for compensation in cognitive neuroscience: increased activity in the previously active regions, recruitment of more brain regions, or engaging both hemispheres where only one was previously activated. In addition, Cappell et al. (2010) suggested that certain brain regions might engage sooner in older adults to compensate to meet demands even when cognitive load is low. While our study focuses on aspects of the parasympathetic cardio regulation, there is a good example of compensation in the sympathetic nervous system. Age-degradation leads to deficits in norepinephrine reuptake in the cardio sympathetic neuromuscular junction. When this occurs, there is an increase in expression of pre-junction catecholamines—specifically, an increase in norepinephrine spillover (Kaye and Esler, 2008). The spillover is a shift in the system resulting in more norepinephrine produced within the cells to compensate for the loss at the junction.

In order to detect developmental changes in the association between RSA and cognitive regulation involving potential compensation among older mothers of young children, researchers need to consider not only age differences in average cognitive regulation (which as noted above, shows shifts toward decreases across middle age into old age), but also how various systems of the body supporting cognitive regulation changes in their covariation with each other and in their independent and interactive links with executive function (EF), effortful control (EC), and cognitive regulation (CR). As noted above, RSA and frontoparietal alpha-power coherence change with age. However, to our knowledge no prior studies have tested for age differences in the covariation between RSA and frontoparietal alpha-power coherence, or age differences in RSA and frontoparietal alpha-power coherence statistical predictions of cognitive regulation phenotypes (i.e., EF, EC, CR)—let alone within the context of studying adult development of women raising children.

To address these two major gaps: (Aim 1) we tested whether the covariation between RSA and frontoparietal alpha-power coherence differed by maternal age, and then (Aim 2) tested whether there are additive or interactive statistical predictive effects of RSA and frontoparietal alpha-power coherence on variance in cognitive regulation (i.e., EF, EC, CR) differed by maternal age. Based on SOC theory, we hypothesized that the systems of the body supporting cognitive regulation would show a developmental shift toward compensation spanning early through middle adulthood (e.g., 20 to 50 s). We expected to find significant maternal age statistical moderating effects on the association between RSA and frontoparietal alpha-power coherence (Aim 1), and on the additive or interactive statistical effect of RSA and frontoparietal alpha-power coherence on EF, EC, and CR (Aim 2). Given that the current study is the first of its kind, we did not have specific expected results regarding the exact age-based patterns in the results from Aim 1 and Aim 2 analyses. However, based on related research, we expect cognitive regulation to be somewhat automated in adulthood, and because of that, we expect cardiac and frontoparietal function to be specialized and not tightly coupled. This idea is based on shifts seen between early development—where cardiac regulation and frontoparietal coherence are tightly coupled when predicting good cognitive function—and young adults where there is less coupling (Sauseng et al., 2005). We further anticipate that the system might be starting to decline in the older women, so there might be more coupling between the cardiac and frontoparietal functions as those systems work together to maintain optimum levels of cognitive regulation. This expectation is based on the decrease in RSA with age and the association between RSA and cognitive regulation as well as the association between frontoparietal coherence and cognitive regulation.

## 2. Materials and methods

### 2.1. Participants

The sample included 90 women (age, *M* = 32.35 years, *SD* = 5.86 years, *range* = 21–49 years). The original sample size was 127 women who had some physiological and behavioral data, but 26 participants were missing one or more of the key variables needed for our analyses, and another 11 were excluded because their ECG data showed an unexplainable decrease in heart rate when shifting from resting state to active cognitive task states (for more details see Deater-Deckard and Bell, 2017). When we compared the included and excluded sub-samples on key demographic measures (e.g., mother and father years of education), there were no significant differences (e.g., two-tailed *t*-test *p*-values greater than 0.10).

There are multiple viewpoints regarding the definition of “woman”; in the current study, we included self-identifying women. Families resided in rural, small town and small city areas of southwest Virginia on the eastern edge of the Appalachian region of the United States. The racial background of these individuals reflected the distribution in the region (74% Caucasian, 13% African American, and 2% Asian, 6% multiracial, 5% other). Regarding the education level of the mothers, 22% had a high school diploma/graduate equivalent diploma (GED) or less; 28% had some college or an associate degree; 30% had a 4-year degree; and 20% had a postgraduate degree.

### 2.2. Procedures

One-third of the participants were recruited as part of a longitudinal study exploring mother-child interactions (children were 3-years old). The other two-thirds were recruited via advertisements and flyers distributed to community organizations also as a part of a family, community-based research project (children were 3 to 7-years old). Signed consent was obtained as part of an informed consent procedure during the laboratory visit. Participants also completed demographic and emotional regulation questionnaires which were submitted in person or by mail after the laboratory visit. Physiological measures were collected during resting conditions (eyes opened and eyes closed paradigm) for 2 min total, and continuously during task conditions involving executive function tasks. An honorarium was given for participation. IRB approval was granted through Virginia Polytechnic Institute and State University.

### 2.3. Measures

#### 2.3.1. Executive function (EF)

Four counterbalanced tasks that measured attentional control, inhibitory control, and working memory were administered on a computer or face to face.

##### 2.3.1.1. Tower of Hanoi

This task was a computerized adaptation of the classic peg task or game. Three disks were all placed on one of three pegs in order from largest to smallest. The participants were required to end with all the pegs in the same order on one of the other pegs. While transferring the pegs, a disk placed on top can never be larger than the one below. The score was measured in seconds to finish with a 60 s maximum possible score (Davis and Keller, 1998).

##### 2.3.1.2. Backward digit span task

Participants heard a set of digits (0–9) that they then repeated back in reverse order. After two practice trials (two-digit list), the test began with an increasing number of digits (two attempts permitted per series of digits). Each correct trial was followed by a subsequent trial increasing the number of digits by one digit. The final score was the longest series of correctly reported digits (i.e., span).

##### 2.3.1.3. Wisconsin card sorting test (WCST)

Participants were shown four cards with different symbols that varied in quantity, shape, and color. They then had to match a stack of 64 or 128 cards (depending on test laboratory site), first by detecting the matching rule (match on quantity, shape, or color) through trial and error, then continuing to match on that rule until the rule changed without warning. At a rule change, the participant would then need to try a new matching rule, and this continued with rule changes throughout the task. The score for analyses was the number of perseveration errors (i.e., the number of continuing to use the old rule after a rule change) (Heaton and Staff, 2003).

##### 2.3.1.4. Stroop color-word task

This task was a computerized version of the classic Stroop color-word task (Stroop, 1935). The name of a color is written in either the matching color (congruent) or a different color (incongruent). The participant indicated the color of the letters using keyboard presses. After several practice trials, the participant completed 20 trials each of congruent, incongruent, and mixed congruent/incongruent. The score was the number of correct responses in the most difficult mixed congruent/incongruent trial block.

##### 2.3.1.5. Executive function composite score

The individual task performance scores described above were compiled into a general EF composite to capture the most reliable general EF performance measure (Miyake and Friedman, 2012). The first principal component among the four task scores (with Stroop, Wisconsin Card Sorting Test, and Tower of Hanoi scores reversed so that higher scores represented better performance) explained 41% of the variance (loadings from 0.57 to 0.75). Indicators were standardized, averaged, and standardized again to compute a composite z-score.

#### 2.3.2. Effortful control (EC)

Participants completed the Adult Temperament Questionnaire Short Form (Evans and Rothbart, 2007), with items rated on a seven-point Likert scale (1 = strongly disagree to 7 = strongly agree). We used the Effortful Control Scale score that comprises inhibitory control, attention control, and activation control and represents self-reported cognitive regulatory capacity (α = 0.68).

#### 2.3.3. Cognitive reappraisal (CR)

Mothers completed the Emotional Regulation Questionnaire (Gross and John, 2003) that includes items rated on a seven-point Likert scale. We used the Cognitive Reappraisal Scale (α = 0.81), which captures self-reported frequency of use of cognitive strategies for reappraising events and feelings to mitigate the effects of negative emotional states. This scale captures self-construed utilization of reappraisal, not accuracy or effectiveness of strategy use.

#### 2.3.4. Respiratory sinus arrhythmia (RSA)

Electrocardiography was used to derive RSA. Two disposable ECG electrodes were placed by the participants themselves with help and instruction from research assistants on the right collarbone and lower left rib cage (Stern et al., 2001). A ground lead was positioned near the base of the scalp. Raw cardiac measures were amplified with a James Long Bioamp (Caroga Lake, NY, USA) with a bandpass from 0.1 to 100 Hz. The data were digitized at 512 samples per second with Snapshot-Snapstream analyzation software (HEM Data Corp., Southfield, MI, USA). ECG signals were manually checked to remove any potentially erroneous R peaks. A four-pass peak detection algorithm was used to identify the R-waves which were then calculated for inter-beat-interval (IBI). Respiratory sinus arrhythmia was derived from the IBI as the high-frequency heart rate variability.

Aggregated continuous physiological scores were derived within each condition state (e.g., resting, task 1, task 2, etc.). Principal components analysis (PCA) of these indicators showed that the first component accounted for 89% of the variance, with loadings from 0.92 to 0.95. The resting and task state indicators of RSA were averaged and then standardized to center the scores for subsequent statistical analyses.

#### 2.3.5. Frontoparietal alpha-power coherence

Frontoparietal alpha-power coherence was collected using EEG. Alpha-power measures were acquired with the International 10–20 System consisting of 16 electrodes per hemisphere and were recorded with an Electro-Cap (Eaton, OH, USA) following standard guidelines. The James Long Bioamp (Caroga Lake, NY, USA) was used for the amplification of the electrical signals with pass from 1 to 100 Hz while impedance was kept below 10 K ohms. The data was then analyzed with EEG Analysis System software (James Long Company; Caroga Lake, NY, USA). Artifacts from eye and gross motor movements were eliminated from analysis. Cleaned data were converted to Hamming windows (1 s) with a 50% overlap and then were transformed with discrete Fourier processing. The alpha band power was computed for 8–13 Hz expressed as mean square microvolts. Transformation for normal distribution was achieved with a natural log transformation. Frontoparietal alpha-power coherence was calculated as the squared correlation between the frontal and parietal alpha-power F3, F4, P3, P4, P7, and P8 sites using an algorithm by Saltzberg et al. (1986), Equation 9.

As with RSA, aggregated continuous physiological scores were derived within each condition state (e.g., resting, task 1, task 2, etc.). PCA was again used to confirm the reliability of these composites. The first component for each composite: F3P3, explained 65% of the variance with loadings from 0.80 to 0.82; F3P7, 61%, loadings 0.78 to 0.80; F4P4, 71%, loadings 0.83 to 0.86; and F4P8, 66%, loadings from 0.79 to 0.83. A composite F3-P3/F3-P7 frontoparietal coherence value was derived from averaging the F3P3 and F3P7 sites, and a composite F4-P4/F4-P8 frontoparietal coherence value was derived from averaging F4P4 and F4P8 sites. These areas represent the dorsolateral and ventrolateral prefrontal cortexes as well as the posterior parietal cortex. Scores were then standardized to center variables for subsequent analyses.

### 2.4. Data analysis

Descriptive statistics, bivariate correlations, and multiple regression analyses were computed using *IBM SPSS Statistics* (Version 26, 2019). Variables were centered for regression equations and estimation of potential statistical interaction effects. *Post hoc* probing of significant interaction terms was conducted using analysis of simple slopes. For Aim 1, we sought to determine whether maternal age moderated the association between RSA and frontoparietal alpha-power coherence. To this end, we used multiple regression to test the statistical prediction of RSA from frontoparietal alpha-power coherence and maternal age (and their interaction); and the statistical prediction of frontoparietal alpha-power coherence from RSA and maternal age (and their interaction). For Aim 2, we used multiple regression to test whether maternal age moderated the independent or interactive statistical predictive effects of RSA and frontoparietal alpha-power coherence on cognitive regulation measures (i.e., EF, EC, CR).

## 3. Results

Descriptive statistics and bivariate correlations (see Table 1) were calculated. All variables were normally distributed (skewness from 0.03 to 0.48) and showed modest or moderate kurtosis (−0.51 to 0.97). Regarding frontoparietal alpha-power coherence, all frontoparietal sites had similar means and standard deviations. Turning to bivariate correlations, EC was positively associated with CR, as well as with lower frontoparietal alpha-power coherence at F3-P3/F3-P7. Frontoparietal alpha-power coherence of the two hemispheres (F3 and F4 sites) was positively correlated. Age was positively associated with EF, and negatively associated with RSA and frontoparietal alpha-power coherence at F4-P4/F4-P8.

**TABLE 1:** Descriptive statistics and bivariate pearson correlations.

		EF	CR	EC	RSA	FP_C_^F3^	FP_C_^F4^	Age
EF		–						
CR		0.00	–					
EC		0.04	0.40**	–				
RSA		−0.02	0.18^+^	0.07	–			
FP_C_^F3^		−0.03	−0.15	−0.24*	−0.19^+^	–		
FP_C_^F4^		−0.11	−0.10	−0.14	0.01	0.50***	–	
Age (years)		0.23*	0.11	0.12	−0.26	−0.10	−0.30**	–
	Mean	0.07	5.01	4.52	3.01	0.14	0.14	32.35
	SD	1.03	0.99	0.75	0.63	0.03	0.03	5.86

Two-tailed *p*-values: ^+^*p* < 0.10, *P < 0.05, **P < 0.01, ***P < 0.001. EF, executive function; CR, cognitive reappraisal; EC, effortful control; RSA, respiratory sinus arrhythmia; FPc-F3, frontoparietal coherence for F3-P3/F3-P7 sites; FPc-F4, frontoparietal coherence for F4-P4/F4-P8 sites.

**FIGURE 1:**
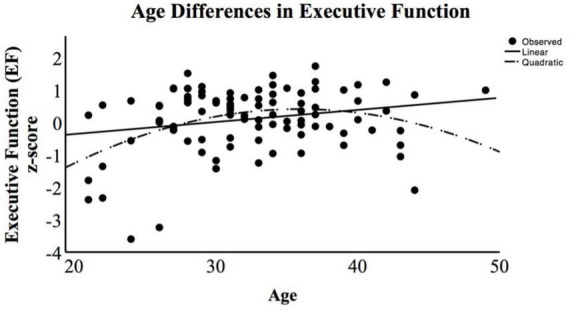
Age (*x*-axis) association with executive function (EF) composite z-score (*y*-axis). This scatterplot includes the linear and quadratic functions representing the association between maternal age and EF task performance, indicating a negative parabolic function with the apex of performance occurring in the mid-30 s with a decrease that continues through the older half of the sample.

For descriptive purposes, we also examined whether the age differences in EF replicated the cross-sectional pattern found in prior studies. That prior evidence indicates a positive linear association between age and general EF in community samples of adults from 20 to 60 years (e.g., Yao et al., 2020), as well as a negative parabolic (i.e., inverted “u”) quadratic growth pattern spanning adulthood. We examined our data and found this pattern, as shown in Figure 1. Executive function task performance peaks in the 30 s and then begins a gradual decline across the 40 s (in the current study data) and, as noted in prior literature, continues to decline across the 50 s and onward (e.g., LaPlume et al., 2022).

We also examined potential curvilinear age-based functions in EC and CR, but did not see significant non-linear functions. For EC, *F*_(2,94)_ = 1.176, *p* = 0.313, *R*^2^ = 0.024; for CR, *F*_(2,91)_ = 0.025, *p* = 0.975, *R*^2^ = 0.001. We also estimated curvilinear age-based functions for RSA *F*_(2, 91)_ = 5.380, *p* = 0.006, *R*^2^ = 0.106; frontoparietal alpha-power coherence F3-P3/F3-P7 *F*_(2, 88)_ = 1.117, *p* = 0.332, *R*^2^ = 0.025; and frontoparietal alpha-power coherence F4-P4/F4-P8 *F*_(2, 88)_ = 4.503, *p* = 0.014, *R*^2^ = 0.093. Both RSA and F4-P4/F4-P8 showed an age-based decrease that decelerated (i.e., “flattened out”) with age.

### 3.1. Aim 1 results

Our first aim was to test whether the covariation between RSA and frontoparietal alpha-power coherence differed by maternal age. We started by estimating four equations (one for each frontoparietal site combination) with the statistical predictors of age, frontoparietal alpha-power coherence, and their two-way interaction term and RSA as the dependent variable. For a thorough analysis, we estimated the four equations a second time, swapping the position of RSA and frontoparietal alpha-power coherence—that is, with the predictors of age, RSA, and their two-way interaction term and frontoparietal alpha-power coherence as the dependent variable.

#### 3.1.1. F3-P3/F3-P7 and RSA

When we tested age and frontoparietal alpha-power coherence for F3-P3/F3-P7 as predictors of RSA, the equation was significant: *F*_(3, 86)_ = 4.378, *p* = 0.006, *R*^2^ = 0.132. However, when we tested age and RSA as predictors of frontoparietal alpha-power coherence for F3-P3/F3-P7, the equation was not significant: *F*_(3, 86)_ = 2.542, *p* = 0.062, *R*^2^ = 0.081. Because this equation was not significant when estimated both ways, we did not proceed with an interpretation involving F3-P3/F3-P7 for Aim 1.

#### 3.1.2. F4-P4/F4-P8 and RSA

When we tested age and frontoparietal alpha-power coherence for F4-P4/F4-P8 as predictors of RSA, the equation was significant. The equation also was significant when examining age and RSA as predictors of frontoparietal alpha-power coherence for F4-P4/F4-P8. Since this equation was significant when estimated both ways, we proceeded with interpretation. Full results are shown in Table 2.

**TABLE 2:** Aim 1 regression results for age as moderator of association between frontoparietal coherence (for F4-P4/F4-P8) and RSA.

Age as moderator of FPc predicting RSA					Age as moderator of RSA predicting FPc				
***F*_(3, 86)_ = 4.515, *p* = 0.005, *R*^2^ = 0.136**					***F*_(3, 86)_ = 4.905, *p* = 0.003, *R*^2^ = 0.146**				
	**B**	**(se)**	**β**	***p***		**B**	**(se)**	**β**	***p***
Age	−0.300	(0.101)	−0.315	0.004	Age	−0.339	(0.109)	−0.326	0.003
FPc	−0.051	(0.098)	−0.055	0.606	RSA	−0.028	(0.117)	−0.026	0.811
Age × FPc	0.190	(0.088)	0.219	0.033	Age × RSA	0.190	(0.101)	0.194	0.062

FPc, frontoparietal coherence; RSA, respiratory sinus arrhythmia.

**FIGURE 2:**
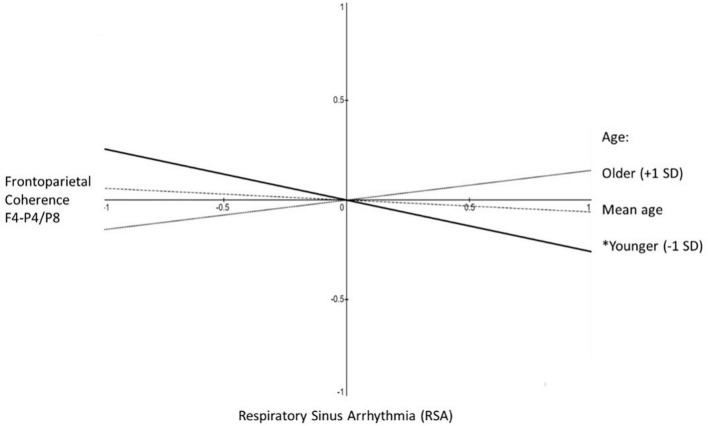
Aim 1 simple slopes. Shown are the simple slopes reflecting the statistical prediction of frontoparietal coherence (F4-P4/F4-P8) from RSA as a function of maternal age. Slopes are shown at one standard deviation below mean age (solid line, *p < 0.05), at mean age (dashed line, non-significant slope), and one standard deviation above mean age (dotted line, non-significant slope).

The two-way interactions involving age were significant or marginally significant (depending on the equation). To interpret this age statistical moderation effect, we estimated simple slopes with age as the moderator of the association between frontoparietal alpha-power coherence for F4-P4/F4-P8 and RSA. With age as the moderator in the equation frontoparietal alpha-power coherence predicting RSA the results indicated −2 standard deviations below mean age, β = −0.47, *p* < 0.01; −1 SD, β = −0.26, *p* < 0.05; at mean age, β = −0.06, n.s.; + 1 SD above mean age, β = 0.15, n.s.; at + 2 SD, β = 0.36, n.s. (see Figure 2). For completeness, we also examined age as the moderator in the equation RSA predicting frontoparietal alpha-power coherence and found a very similar pattern: −2 standard deviations below mean age, β = −0.37, *p* < 0.06; −1 SD, β = −0.20, n.s.; at mean age, β = −0.06, n.s.; + 1 SD above mean age, β = 0.15, n.s.; at + 2 SD, β = 0.32, n.s. The only significant association observed between frontoparietal alpha-power coherence and RSA was a negative association that was observed only among younger mothers.

### 3.2. Aim 2 results

Our second aim was to test for additive or interactive statistical predictive effects of RSA and frontoparietal alpha-power coherence on variance in EF, EC, and CR as a function of maternal age.

#### 3.2.1. EF, RSA, and F3-P3/F3-P7

The equation for EF with the additive and interactive effects of age, RSA, and frontoparietal alpha-power coherence for the F3-P3/F3-P7 composite was not significant: *F*_(7, 82)_ = 1.648, *p* = 0.134. We did not proceed with further analysis involving F3-P3/F3-P7.

#### 3.2.2. EF, RSA, and F4-P4/F4-P8

The equation for EF with additive and interactive effects of age, RSA, and frontoparietal alpha-power coherence for the F4-P4/F4-P8 composite was significant. Results are shown in Table 3. To interpret the age differences in RSA and frontoparietal alpha-power coherence moderation effects in the statistical prediction of EF, we estimated simple slopes separately for younger and older women (median split on age), then computed simples slopes to interpret (1) the association between frontoparietal alpha-power coherence for F4-P4/F4-P8 and EF with RSA as a moderator, and (2) the association between RSA and EF with frontoparietal alpha-power coherence as the moderator.

**TABLE 3:** Aim 2 regression results for age as moderator of association between frontoparietal coherence (for F4-P4/F4-P8) and RSA predicting executive function.

Age, FPc, and RSA predicting EF				
***F*_(7, 89)_ = 3.094, *p* = 0.006, *R*^2^ = 0.141**				
	**B**	**(se)**	**β**	***p***
Age	0.193	(0.113)	0.190	0.092
FPc	−0.094	(0.109)	−0.097	0.388
RSA	−0.115	(0.130)	−0.109	0.337
Age × FPc	0.268	(0.105)	0.290	0.012
Age × RSA	−0.013	(0.121)	−0.013	0.916
FPc × RSA	0.023	(0.119)	0.024	0.849
Age × FPc × RSA	−0.211	(0.086)	−0.303	0.016

FPc, frontoparietal coherence; RSA, respiratory sinus arrhythmia.

**FIGURE 3:**
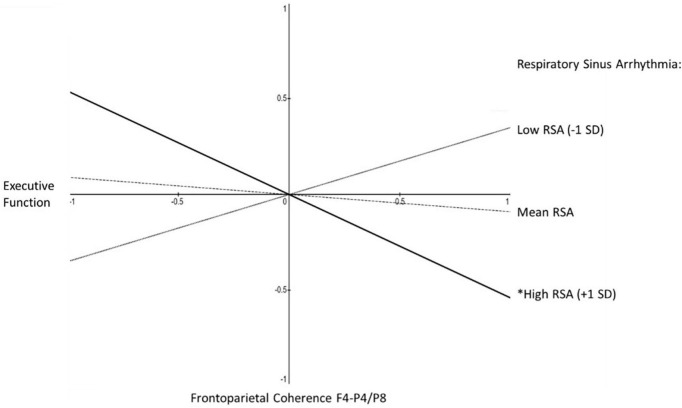
Aim 2 simple slopes, for older half of sample. Shown are the simple slopes for the older half of the sample, reflecting the statistical prediction of executive function from frontoparietal coherence (F4-P4/F4-P8) as a function of respiratory sinus arrhythmia (RSA). Slopes are shown at one standard deviation above mean RSA (solid line, *p < 0.05), at mean RSA (dashed line, non-significant slope), and one standard deviation below mean RSA (dotted line, non-significant slope). In contrast, none of the simple slopes was significantly different from 0 among the younger half of the sample.

A clear age-difference pattern emerged in the simple slope estimates. For the younger half of the sample, there was not a single significant simple slope for the association between EF and either RSA or frontoparietal alpha-power coherence in either equation (i.e., with RSA as moderator of frontoparietal alpha-power coherence predicting EF, or with frontoparietal alpha-power coherence as moderator of RSA predicting EF). In contrast, there was emergence of significant simple slope estimates among the older half of women in the sample. For RSA as moderator of frontoparietal alpha-power coherence predicting EF the results indicated −2 standard deviations below mean RSA, β = 0.80, *p* < 0.05; −1 SD, β = 0.35, n.s.; at mean RSA, β = −0.09, n.s.; + 1 SD above mean RSA, β = −0.54, *p* < 0.05; at + 2 SD, β = −0.98, *p* < 0.05 (see Figure 3). For frontoparietal coherence as moderator of RSA predicting EF: −2 standard deviations below mean frontoparietal coherence, β = 0.59, *p* < 0.05; −1 SD, β = 0.25, n.s.; at mean RSA, β = −0.09, n.s.; + 1 SD above mean RSA, β = −0.44, n.s.; at + 2 SD, β = −0.78, *p* < 0.06.

#### 3.2.3. EC and CR

In contrast to the analyses for EF, none of the equations for EC and CR were significant; *F* statistics ranged from 1.33 to 1.70, with *p*-values ranging from 0.121 to 0.246. However, the significant three-way interaction effect (F4-P4/F4-P8 frontoparietal alpha-power coherence × RSA × age) that we found for EF was of similar effect size and direction and approaching significance for EC (*p* < 0.08). Also, EC and CR were correlated 0.40, suggesting a potential general self-perceived cognitive regulation indicator. Thus, in a *post hoc* analysis, we averaged the EC and CR scores (standardized) and estimated the equation for Aim 2 again using the composite EC-CR score as the dependent variable. The equation for F4-P4/F4-P8 was significant: *F*_(7, 79)_ = 2.20, *p* = 0.043, *R*^2^ = 0.16, and the three-way interaction term was marginally significant, β = −0.25, *p* = 0.057. Analysis of simple slopes showed a pattern that had some similarity to the pattern for EF. Among older women only, F4-P4/F4-P8 frontoparietal alpha-power coherence was a significant negative statistical predictor of EC-CR at higher levels of RSA (+ 1 SD, β = −0.60, *p* < 0.02; + 2 SD, β = −0.83, *p* < 0.04). Also, among older women only, RSA was a significant positive statistical predictor of EC-CR at lower levels of F4-P4/F4-P8 frontoparietal alpha-power coherence (−1 SD, β = 0.44, *p* < 0.001; + 2 SD, β = 0.73, *p* < 0.003).

We ran *post hoc* simple slopes analyses for Aims 1 and 2 during *task only* to clarify whether the effects were driven by general RSA levels, or specifically the levels during cognitive tasks. All effects were in the same direction but were smaller than the resting/task averages results. For Aim 1 F4 composite predicting RSA moderated by age the effect sizes were + 2 SD β = 0.28, + 1 SD β = 0.11, −1 SD β = −0.25, −2 SD β = −0.42 and for Aim 2 Older moms F4 composite predicting EF moderated by RSA effect sizes were + 2 SD β = −0.64, + 1 SD β = −0.33, −1 SD β = 0.29, −2 SD β = 0.60.

## 4. Discussion

The broad goal of the current study was to examine a potential developmental shift from optimization to compensation across adulthood (Baltes, 1997; Freund and Baltes, 1998) in mothers’ system of cognitive regulation involving various physiological, behavioral, and survey-based indicators. These are key physiological aspects of well-regulated, non-reactive maternal parenting behavior that is instrumental to warm, sensitive and responsive caregiving (e.g., Crandall et al., 2015; Deater-Deckard and Bell, 2017). We had two primary aims. For Aim 1, we tested potential developmental changes in how cardiac and cerebral physiological indicators of cognitive regulation covaried with each other as part of an integrated system. Results indicated an age difference in how frontoparietal alpha-power coherence and RSA covaried, though only for the F4-P4/F4-P8 composite.

Specifically, the results from Aim 1 indicate there was a significant negative association that dissipated with age. For younger mothers, having higher RSA predicted lower frontoparietal alpha-power coherence with the same pattern when frontoparietal alpha-power coherence predicted RSA—an association that did not hold for older mothers in the sample. RSA and frontoparietal alpha-power coherence both are considered as important indicators of the efficiency and effectiveness of regulatory capacity (for RSA, Geisler et al., 2013; Wang et al., 2013; Balzarotti et al., 2017; for frontoparietal coherence, Klimesch et al., 1999; Bell, 2001; Moore et al., 2008). A preliminary interpretation of the cross-sectional age difference in the current study is that it reflects the specialization between frontoparietal alpha-power coherence and RSA which was previously seen in adolescence and early adulthood samples. This may indicate that optimization is denoted by specialized activity of those two systems followed by a developmental shift toward less negative coupling. This is a pattern that may reflect a shift away from optimization toward a compensatory process involving greater flexibility in utilization of frontoparietal alpha-power coherence and RSA. This may represent the kind of compensatory process proposed by SOC theory (Baltes, 1997). A competing interpretation is that the age difference we detected may not reflect a shift from an optimized system toward a compensating process. They could instead reflect age-based degradation in the system. A fuller understanding of whether the age-difference pattern just described reflects a shift toward compensation requires examination of whether and how frontoparietal alpha-power coherence and RSA work together to account for variation in multiple diverse indicators of maternal cognitive regulation. To that end, in Aim 2, we tested for a potential age difference in the additive and interactive associations of RSA and frontoparietal alpha-power coherence with maternal EF, EC, and CR.

We examined maternal age as a statistical moderator, and the equations examined variance in EF, EC, and CR based on physiological statistical predictors. As with the analyses for the first aim, statistically significant equations were found only for the F4 composite. Furthermore, for the Aim 2 analyses, the equation was significant only for EF, and results indicated a significant three-way interaction effect between age, RSA, and frontoparietal alpha-power coherence. To interpret the statistical interaction, we performed *post hoc* analyses. In *post hoc* analyses, we used age as the first moderator by dividing the sample at the median. We then found significant results when frontoparietal alpha-power coherence was used as the second moderator for RSA predicting EF, *and* when RSA was used as a moderator for frontoparietal alpha-power coherence predicting EF (we also found a similar pattern of this effect for a composite of self-reported EC and CR). Results for Aim 2 indicate that younger women may be using fewer physiological resources while attending to and utilizing relevant information during cognitively demanding tasks. This could suggest that there is more automation in psychophysiological mechanisms in the central and peripheral nervous systems in young adulthood compared to older adulthood (e.g., Cabeza et al., 2018; Hamilton and Allard, 2021). This may mean that their cognitive regulation is not contingent on the physiological components of self-regulation capacity in the way that it is for older mothers.

Among older mothers only, EF performance was associated with both RSA and frontoparietal alpha-power coherence in an interaction effect that may be indicative of compensatory processes. Lower RSA was offset by higher frontoparietal alpha-power coherence to predict higher EF, and higher RSA was offset by lower frontoparietal alpha-power coherence to predict higher EF. This may indicate that among older mothers, frontoparietal alpha-power coherence is able to compensate for poorer autonomic regulation of the heart, and better autonomic regulation of the heart is able to compensate for higher frontoparietal alpha-power coherence (representing less automatic, more effortful processing). Furthermore, this interactive pattern among older mothers also may be present for self-reported indicators of cognitive regulation (including effortful control and cognitive reappraisal), but our findings in this regard are tentative and require replication before interpreting further.

What are the implications of these results regarding harsh reactive vs. well-regulated caregiving? In prior analyses with the same sample (Deater-Deckard and Bell, 2017), we reported that the well-established link in the literature between higher maternal EF and non-reactive supportive parenting may reflect the roles of both cognitive and cardiovascular activity—although those prior results were ambiguous with respect to the precise patterns of regulatory aspects of cognitive and cardiac functioning. The current results suggest that age-based changes in maternal cardiac and cerebral regulation during effortful cognitive processing reflect developmental aging effects, and perhaps compensatory effects within the body, for promoting better cognitive regulation. Better cognitive regulation increases self-regulatory resources for mothers—perhaps especially among older mothers—to enact caregiving behaviors that decrease the frequency and strength of impulsive reactive responses to the acute and chronic stressors that arise when caring for young children (Crandall et al., 2015).

### 4.1. Limitations

The current study is, to our knowledge, the first of its kind and it also has several strengths (e.g., multi-method, wide age range of mothers, examination of multiple indicators of cognitive self-regulation capacity). These strengths aside, the study had several limitations that should be considered. First, the cross-sectional correlational study design limits the interpretation of developmental change and potential causality in the detected statistical associations between frontoparietal alpha-power coherence, RSA, and cognitive regulation measures.

In addition, we did not control for parity or child age (although this did not vary widely in the current sample); addressing these variables may be beneficial for future studies. Furthermore, although the study sample was broadly representative of the region where the families lived, it was not representative of the broader region of the country or the entire country. Given the overall lack of racial and ethnic diversity in the sample, we were not adequately powered to test for potential group differences in our study aims. Relatedly, the current research also would be well complemented by future studies that consider age-based changes in non-maternal women, and paternal and non-paternal men.

Finally, our inferences regarding age-based differences in mothers’ cognitive regulation implicates ovarian aging and other hormonal changes across adulthood, but we did not have measures of self-reported ovarian aging indicators or of hormones. Although some of the older individuals in the sample may be perimenopausal, hormone levels are quite stable within the age range of the current study, with only slight declines on average prior to the onset of menopause (usually between 40 and 44 years) (e.g., Burger et al., 2007; Butler and Santoro, 2011).

Future research could address some of these limitations as well as test for replication and extension of our findings. For example, we will be testing for replication in a second larger cross-sectional study of mothers. In addition to our next study, it will be essential to elucidate within-person changes longitudinally, in the interacting effects of frontoparietal functioning and vagal regulation as related to cognitive regulation among mothers. Future studies could also include direct or indirect measures of ovarian age and menopausal status, to elucidate whether and how those aspects of physiological development may covary with or even explain the age differences we found.

With these caveats and future directions considered, there are several key conclusions to be drawn from the current study. We found that there are age-related differences in the interactions between RSA and frontoparietal alpha-power coherence (Figure 2), and that those differences pertain to cognitive regulation in older mothers (Figure 3). This may reflect developmental shifts toward compensation for aging- and learning-related changes in cognitive regulation among mothers as they raise their children. These age differences probably reflect a gradual yet constantly developing whole-body system of physiological and cognitive self-regulation that is essential for maintaining responsive caregiving in the face of the many challenges of parenting. Also, the separate components of this whole-body system likely do not covary with each other with age, and likely interact with each other in their effects on cognitive regulation. Thus, researchers should not be deterred if they at first observe non-significant zero-order correlations or lack of significant additive statistical predictive effects. Finally, the evidence for developmental changes in this system of regulation will very likely depend on the specific methods and measures used for operationalizing key indicators captured by physiology, behavior, and self-perceptions.

## Author’s note

Data and study materials, and the data analysis syntax, are available upon request to the corresponding author. This study was not pre-registered.

## Data availability statement

The original contributions presented in the study are included in the article/supplementary material, further inquiries can be directed to the corresponding author.

## Ethics statement

The studies involving humans were approved by the Virginia Tech Institutional Review Board. The studies were conducted in accordance with the local legislation and institutional requirements. The participants provided their written informed consent to participate in this study.

## Author contributions

JC and KD-D: data analyses, intellectual contribution, and manuscript preparation. MB: intellectual contribution and manuscript review. All authors contributed to the article and approved the submitted version.
